# Zinc use efficiency is enhanced in wheat through nanofertilization

**DOI:** 10.1038/s41598-018-25247-5

**Published:** 2018-05-01

**Authors:** Ashwin Dapkekar, Paresh Deshpande, Manoj D. Oak, Kishore M. Paknikar, Jyutika M. Rajwade

**Affiliations:** 1 Nanobioscience group, Agharkar Research Institute, G. G. Agarkar Road, Pune, 411 004 India; 2 Genetics and Plant Breeding, Agharkar Research Institute, G. G. Agarkar Road, Pune, 411 004 India; 3 SavitribaiPhule Pune University, Ganeshkhind, Pune 411 007 India

## Abstract

Ferti-fortification of wheat with zinc, an essential micronutrient is one of the strategies for combating ‘hidden hunger’ in a large proportion of people all over the world. During fertilization, application of large quantities of micronutrients often results in nutrient wastage and subsequent environmental pollution. Here, we report zinc complexed chitosan nanoparticles (Zn-CNP) for ferti-fortification of durum wheat in field-scale experiments. The efficacy of Zn-CNP was assessed vis-à-vis conventionally applied ZnSO_4_ (0.2%; 400 mgL^−1^ zinc) in two durum wheat genotypes (MACS 3125, an indigenous high yielding genotype and UC 1114, a genotype containing the *Gpc-B1*gene). The observed grain zinc enrichment using Zn-CNP nanocarrier (~36%) and conventional ZnSO_4_ (~50%) were comparable, despite 10 folds less zinc (40 mgL^−1^) used in the former. Nanofertilizer application increased grain zinc content without affecting grain yield, protein content, spikelets per spike, thousand kernel weight, etc. Grain zinc enrichment observed in the four-year field trials on plots with varying soil zinc content was consistent, proving the utility of Zn-CNP as a novel nanofertilizer which enhanced fertilizer use efficiency. Our work describes a new paradigm in micronutrient fortification, viz. ‘use nanofertilizers at the right place, right time and in right doses’.

## Introduction

Zinc is a vital trace element, essential for human health. Deficiency of Zn is a well-documented public health issue especially in the developing world^1–4^, affecting approximately one-third of the population around the globe especially children and pregnant women. Zinc deficiency can lead to impairment of the immune system, physical growth retardation, and reproductive health etc.^5–7^. The primary reason for the occurrence of Zn deficiency in the developing countries in the world appears to be poor dietary diversity (characterized by non-inclusion of pulses, green leafy vegetables, nuts, meat, eggs and animal-derived food) as well as very low intake of dietaryZn^8,9^. Wheat is the staple food in many countries of the world and provides the necessary calories and proteins. However, its micronutrient content is extremely low (e.g., Zn about 20–40 mgkg^−1^). Presence of phytates further lowers the availability of micronutrients such as zinc. In short, the zinc intake is much lower than the daily recommended dietary allowance (10 mg, 12 mg and15 mg for children, adult women and adult men, respectively)^10^. The Zn deficiency manifests in suboptimal health status. For example, in India, a loss of 2.8 million DALYs (disability-adjusted life years) per annum is attributable to Zn deficiency^2^. Thus, production and consumption of zinc-enriched cereals such as wheat could be the most appropriate weapon to fight ‘hidden hunger’ (of micronutrients)^11^.

The soil micronutrient deficiency not only limits the productivity of crops but also lowers grain nutritional quality^12–15^. The ever-declining global soil quality thus poses a formidable challenge to improving grain zinc content and is a high-priority research area^16,17^. To this end, several international programs have been initiated. For example, under the aegis of CGIAR (Consultative Group on International Agricultural Research), a global agriculture research partnership for a food secure future, the HarvestPlus program was initiated in 2003 with the aim of producing micronutrient-rich staple foods to combat micronutrient deficiencies^4^. The HarvestPlus initiative promotes ‘biofortification’ as a preferred strategy for micronutrient enrichment of grains^18^. Research programs directed towards developing high yielding hybrid varieties of crops has increased productivity and production of cereals such as wheat in India^19^. However, no Zn-rich cultivar is yet available, probably because the grain yield and Zn concentration show an inverse correlation. Thus, the prospects of genetic biofortification appear to be bleak^20–24^. Therefore, agronomic biofortification through Zn fertilization (also referred to as ferti-fortification) becomes a method of choice to increase grain Zn content (and possibly yield). In fact, in the event of obtaining genotypes with high grain zinc content in future, agronomic biofortification would be necessary and relevant especially to their cultivation in zinc-poor soils. Thus, agronomic and genetic biofortification could be the most effective complementary approach to obtain zinc- enriched wheat^9,11^.

Agronomic biofortification of wheat using soil, foliar or soil + foliar application of zinc-containing fertilizersis well reported^25–30^. Several inorganic and chelated forms of Zn are useful as fertilizers. Their use efficiency is high in soils with good drainage, favorable pH (slightly acidic to neutral), and adequate organic content. In the absence of these conditions, the fertilizers are wasted, contributing to environmental pollution. Therefore, it is necessary to explore newer approaches for ensuring delivery of fertilizer in ‘right doses’ at the ‘right time’ in a ‘plant-available form’ without causing environmental concerns.

We developed Zn-complexed chitosan-TPP nanoparticles (Zn-CNP, containing ~40 mg/L zinc) and assessed its applicability for biofortification, in durum wheat. In pot experiments, we observed 27 and 42% zinc enrichment in grain in two genotypes, viz., MACS 3125 and UC 1114. The stomatal uptake of Zn-CNP and subsequent zinc accumulation in grain was demonstrated^31^. Gene expression analysis was performed to understand the genes involved in the transport of zinc applied in the form of Zn-CNP vis-à-vis the conventionally used form, i.e., ZnSO_4_. The study indicated involvement of *Gpc-B1* locus and association of zinc with gamma gliadins in the grain endosperm^32^.

The goal of agronomic biofortification is to achieve an improvement in nutritional characteristics without compromising crop yield for which, field-level studies are mandatory. Extending our previous work, we have now assessed robustness and efficacy of our nanoformulation in field-scale plots (differing in soil zinc content) during four consecutive wheat growing seasons. Two durum wheat genotypes/cultivars were tested, viz., MACS 3125, an indigenously developed high-yielding durum wheat cultivar and UC 1114, which is a durum wheat containing the *GpcB1* gene, representing high grain protein. Further, we assessed the effect of different doses of Zn-CNP nano-fertilizer with urea because the latter may improve the effectiveness of foliar-applied Zn ultimately leading to grain Zn enrichment^33^.

## Results and Discussion

During the experimental period during all the four years, minimal variations in the climatic conditions at the location of the trials were recorded (see Supplementary Fig. 1). The data obtained in the 4-year field trials were pooled and analyzed for the effect of (a) different foliar treatments, (b) wheat genotypes and (c) year of the trial, on the grain characteristics.

Considering the treatments, genotypes and the number of trials, a 3-way ANOVA was performed which could give the contribution of the sources individually and due to their interactions to the observed variations in the grain characteristics. The results of three-way analysis of variance (Table 1) showed that the genotypes tested, the foliar treatments and the year in which the experiment was undertaken had a significant effect on the grain characteristics. The latter assumes importance because all environmental factors (zinc content of the soil, radiation for that year, rain, daily maximum and minimum temperatures, relative humidity, cloudy days, etc.) will contribute to the observed effects. In the three-way ANOVA grain Fe content and grains per spike showed highly significant (p < 0.001) interactions of genotype × treatment × year. While significance was reduced for spikelets per spike (p < 0.01) and grain zinc content (p < 0.05). In the three way ANOVA year × treatments interaction were highly significant (p < 0.001) for zinc content, grains per spike; while significance was reduced for grain protein content, spikelets per spike (p < 0.01) and spike length (p < 0.05). Treatments × genotype interactions were significant only in grains per spike and spikelets per spike (p < 0.05 and p < 0.01 respectively). While ‘year × genotype’ interactions were highly significant (p < 0.001) in most of the traits analyzed, they were less significant (p < 0.05) in ‘grain protein content’ and non-significant in ‘spikelets per spike’. The interactions were statistically non-significant for rest of the studied traits. Generally, if interactions were significant, one would ignore the impact of individual components. However, for better understanding, the data were analyzed for individual factor effects also. Individually all the three factors affected the zinc content, protein content, iron content and spike length in a statistically significant manner (p < 0.001). Hence it was appropriate to analyze the response of each factor individually.

**Table 1:** Contributions^$^ due to genotype, trial # (year), foliar treatments on grain characteristics.

Source	Zn (µg g^−1^)	Fe (µg g^−1^)	PC (%)	TKW (g)	Yield (Kg ha^−1^)	Spike length (cm)	GRPS (nos)	Spikelets/spike (nos)
Genotype (G)	01.3***	31.9***	43.4***	54.6***	31.1***	04.8***	00.0 ns	0.01 ns
Trial year (P)	58.5***	42.8***	13.6***	00.5 ns	17.5***	36.8***	10.7***	62.3***
Treatments (T)	29.0***	01.8***	09.5***	00.7 ns	01.2 ns	06.9***	29.5***	01.1 ns
G*P	01.3***	06.1***	02.6**	12.0***	19.1***	06.5***	03.2***	00.9 ns
T*P	02.2***	00.7 ns	05.7**	02.7 ns	03.2 ns	06.8*	18.4***	05.6**
G*T	00.2 ns	00.7 ns	00.7 ns	00.8 ns	00.4 ns	01.5 ns	04.4***	02.7**
G*T*P	01.3*	04.4***	00.9 ns	01.7 ns	01.5 ns	05.4 ns	13.4***	05.3**
Error	06.2	11.6	23.5	27.0	25.9	31.3	20.5	22.0

^$^% of total sum of squares from 3-way ANOVA, ***p < 0.001, **p < 0.01, *p < 0.05 and ns denotes not significant. PC denotes protein content, TKW denotes thousand kernel weight, GRPS denotes grains per spike.

Data presented in Table 2 show that all types of foliar treatments resulted in a statistically significant (p < 0.05) increase in the grain zinc content as compared to control treatment T0 (with water). With T2 the grain zinc content was 59.4 µg g^−1^, which was markedly higher than T3 (46.6 µg g^−1^), T4 (53.3 µg g^−1^), and T5 (43.5 µg g^−1^). Despite the application of low doses of Zn (Zn-CNP, 40 mg L^−1^ Zn), an increased grain zinc content was observed consistently at a 10 fold lower zinc concentration in comparison to conventional fertilizer, ZnSO_4_ (containing 400 mg L^−1^ Zn). It has been reported that Zn concentration in wheat grains can be improved by foliar application^15^. Further, the zinc levels in the grains increase with the application rates of foliar Zn^17^. Our results match well with these findings. According to recent reports, increasing the nitrogen (N) supply enhances grain Zn and Fe concentrations^33,34^. Also, Zn and N applications have a synergistic effect on grain Zn concentration of durum wheat^35^. We observed a statistically significant increase in grain Zn in plants receiving T1 (42.9 µg g^−1^) confirming the role of nitrogen in enhancing the grain Zn. Our observations also establish that application of N fertilizers (in the form of urea) promote uptake and translocation of micronutrients^36–38^.

**Table 2:** Effect of different foliar treatments on the grain characteristics.

Treatment	Description	Zn (µg g^−1^)	Fe (µg g^−1^)	PC (%)	TKW (g)	Yield (Kg ha^−1^)	Spike length (cm)	Grain per spike (nos)	Spikelets per spike (nos)
T0	Control	39.50A	51.36A	16.60A	40.402A	4628A	7.48 B	41.0C	17.4A
T1	Urea	42.86B	54.49B	17.52B	41.029A	4565A	7.22A	38.2A	17.2A
T2	U + ZnSO_4_ (Zn 400 mgL^−1^)	59.40E	54.35B	18.07C	40.677A	4343A	7.39B	37.2A	17.4A
T3	U + ZnSO_4_ (Zn 40 mgL^−1^)	46.62C	52.85A	17.11A	42.202A	4430A	7.21A	37.5A	17.6A
T4	U + Nano-1 (Zn 40 mgL^−1^)	53.30D	55.32B	18.15C	41.721A	4701A	7.33B	39.0B	17.6A
T5	U + Nano-2 (Zn 4 mgL^−1^)	43.50B	51.28A	17.13B	41.468A	4577A	7.19A	40.8C	17.4A
	SE (N = 32)	0.772	1.008	0.187	0.892	139.4	0.53	0.389	0.144
	5% LSD 179 DF	**2**.**155**	**2**.**813**	**0**.**523**	**2**.**490**	**388**	**0**.**148**	**1**.**085**	**0**.**4039**

Values indicate means of 2 genotypes × 4 replicates × 4 trials (years). Means with the same letter are similar at 5% significance level using Fischer’s LSD. LSD values are in bold font to differentiate from data values. PC denotes protein content; TKW denotes thousand kernel weight.

The grain iron content was higher in plants of the T1, T2 and T4 treatments. Grain protein content showed a statistically significant increase after treatments T2 and T4 (18.0 and 18.2 µg g^−1^ respectively) followed by T1 and T5. The grain mineral content is known to be associated with increased protein content^33,39^. We observed that Zn and Fe were positively correlated (Table S3) with grain protein which is similar to earlier reports where the application of Zn-containing fertilizer led to an increase in the Fe, Mn, and protein concentration in wheat^40,41^. In fact, grain protein is a sink for Zn and Fe^17,34^. Higher grain protein is the result of higher Zn concentration as indicated by T2 and T4 (Table 2).

When the crop was harvested at maturity and grain yield calculated, we observed that the overall grain yield [4630 Kg ha^−1^, p < 0.05] was not adversely affected due to the foliar treatments. This finding is in agreement with previous studies which show that crop yield was not affected by the pre-harvest foliar application of minerals^41,42^. The results of this study corroborate the findings that post-flowering applications have a greater impact on grain Zn concentration and a smaller impact on grain yield^17,43^. The use of Zn with urea did not increase the yield or yield components of two wheat genotypes significantly probably because the basal N applied was adequate for wheat production^44^. Foliar treatment did not change grain characteristics such as ‘thousand kernel weight’ and ‘spikelets per spike’, but ‘spike length’ and ‘grains per spike’ were adversely affected. Spike length was shorter in T1, T3, and T5 as compared to T0 (Table 2). According to some of the previous studies, the grain characteristics showed a significant and slightly negative relationship with Zn grain concentration^39,45^. However, Velu *et al*.^46^ did not find any correlation between Zn concentration and grain size. In practice, these data are of little importance for farmers. (For year-wise data, see Supplementary Tables S1 and S2).

In our field-scale trial experiments with Zn-CNP nano-fertilizer, we have chosen the genotypes deliberately, viz., high yield characteristic of the genotype MACS 3125 and high grain protein content of the UC 1114 genotype. To understand the response of the genotypes to the various foliar treatments, we re-analyzed the field trial data collected over 4 years of study. It was observed that (Table 3) in UC 1114 genotype the average zinc, iron and protein concentrations were 48.9 µg g^−1^, 59.8 µg g^−1^, and 18.6% respectively, which were significantly higher than those obtained in MACS 3125 genotype. Whereas, in MACS 3125, the grain yield and thousand kernel weight (TKW) were higher. However, no variations in spike traits such as spike length, grains per spike and spikelets per spike were observed, in both the genotypes. Genotypes/cultivars are known to respond differently to Zn application depending upon their inherent grain Zn density^47,48^. In most cases, grain yield and grain zinc concentration were inversely related^20–23^. UC 1114 showed a late flowering and acceleration in flag leaf senescence as compared to MACS 3125, because of the *Gpc-B1* transcription factor^49^. These traits probably contribute to the observed enhanced grain protein, Zn,and Fe in the UC 1114 genotype. MACS 3125 is a variety obtained by conventional breeding and suited for cultivation in central and peninsular India known for its high yield, hence the results on the grain yield and thousand kernel weight (TKW) were significantly higher regardless of zinc enrichment. Considering the ever-growing global demand for food and wide spread occurrence of zinc malnutrition, increasing grain Zn concentration in high-yielding wheat cultivars is important^50^. Based on the results presented here, foliar application of Zn-CNP1 can be recommended for the biofortification of wheat with Zn without causing any foliar damage and without reducing yield.

**Table 3:** Response of the genotypes to the various foliar treatments with respect to grain characteristics.

Genotype	Zn (µg g^−1^)	Fe (µg g^−1^)	PC (%)	TKW (g)	Yield (Kg ha^−1^)	Spike length (cm)	Grains per spike (nos)	Spikelets per spike (nos)
MACS 3125	46.09A	46.70A	16.25A	46.66 B	5143B	7.39A	39.0A	17.5A
UC 1114	48.97B	59.85B	18.60B	35.83A	3938A	7.21A	39.0A	17.4A
SE (N = 96)	0.445	0.582	0.108	0.515	80.4	0.30	0.224	0.835
LSD (p < 0.05) 179DF	**1**.**244**	**1**.**624**	**0**.**302**	**1**.**437**	**224**.**5**	**0**.**855**	**0**.**626**	**0**.**233**

Values indicate means of 6 treatments × 4 replicates × 4 independent yearly trials. Means with the same letter are similar at 5% significance level using Fischer’s LSD. LSD values are in bold font to differentiate from data values. PC denotes protein content; TKW denotes thousand kernel weight.

As mentioned earlier, the experimental plots chosen the field trials showed variations in soil zinc content. To elucidate the contribution of soil (zinc content and other soil parameters) and the foliar applied fertilizer (T2, T3, T4 and T5) on the zinc biofortification, we tried to examine the data collected at the end of each field trial. In this analysis, we account for variations that exist in the environmental parameters such as maximum and minimum temperatures, radiation received, etc. Here, irrespective of the type of treatment and the cultivar, the data on grain characteristics were averaged and used for the analysis. The average grain zinc and iron content in Trial #4 were the least (Table 4). In trial #3, a marginal increase in the grain zinc was observed which was statistically significant, and the highest grain zinc content was obtained in trials #1 and #2. A good correlation between grain zinc concentration and grain protein content was seen in each of the trials (Table S3). Various environmental factors including soil conditions cause inconsistencies in achieving Zn biofortification^51,52^. This explains the variation recorded in the present field-scale experiment. In both Zn-sufficient and Zn-deficient soils, the foliar ferti-fortification approach is necessary for increasing grain Zn concentration^17,53^.

**Table 4:** The grain characteristics in each field trials.

Field trial	Zn (µg g^−1^)	Fe (µg g^−1^)	PC (%)
#1	50.92C	51.38B	18.14C
#2	61.63D	64.67C	17.69B
#3	40.92B	53.67B	17.52B
#4	36.66A	43.37A	16.35A
SE (N = 48)	0.630	0.823	0.153
LSD (p < 0.05) 179DF	**1**.**759**	**2**.**297**	**0**.**427**

Values indicate means of 6 treatments × 4 replicates × 2 genotypes. Means with the same letter are similar at 5% significance level using Fischer’s LSD. LSD values are in bold font to differentiate from data values. PC denotes protein content.

Correlation coefficients calculated for all the traits (see Supplementary Table S3) indicate a positive correlation between both, grain zinc (r^2^ = 0.49) and iron (r^2^ = 0.71) content with grain protein content. The grain zinc and iron content were positively correlated (r^2^ = 0.64). Over the four field trials, yield and thousand kernel weight showed good correlation (r^2^ = 0.43).

The foliar delivery of zinc-containing fertilizers using nanocarrier represents a novel technology in cereals. The present study establishes enhanced ‘use efficiency’ of zinc upon active uptake, translocation, and accumulation of zinc in the grains. The results obtained in the present study prove that the target zinc concentration in grain was achieved using a fertilizer dose (40 mg L^−1^ Zn) which is 10-fold lower than the recommended dose (400 mg L^−1^ Zn) in case of foliar applied fertilizer. Based on the zinc content of grains, data indicate at an 8-fold higher Zn use efficiency after treatment T4 (in comparison to T2). To the best of our knowledge, this is the first systematic field-scale study on zinc enrichment in wheat using an indigenously developed nano-fertilizer. In a previous study, foliar application of nanoparticulate ZnO resulted in enhanced zinc concentration in maize^54^. Further, the translocation of the zinc in different parts (leaf, cob, and grain) of the maize plant was dependent on the concentration of the ZnO applied. At a lower concentration (100 mg L^−1^ ZnO) the translocation of zinc into grains was maximum whereas, at high concentrations, the zinc was localized in other plant parts^54^. In another study, seed-coating with ZnO nanoparticles (1000 mg L^−1^) has shown positive effects on seed germination and seedling vigor indices, whereas the promotive effects of ZnO nanoparticles were evidenced at relatively low concentrations with foliar application in a field scale study^55^. The grain zinc enrichment achieved in the present study indicates the role of Zn-CNP in making the foliar-applied nutrient in the ‘plant-available’ form and clearly indicating the potential application of nano-fertilizers in agriculture.

## Concluding Remarks

A successful strategy to increase grain zinc content in durum wheat genotypes with inherent high yielding capacity and high protein as developed in the present study is the need of the day, considering the increased global food demand and the problem of zinc malnutrition. Consistent enhancement in grain content observed in the four year trials carried out on plots differing in zinc content indicates robustness of the method and proves the utility of Zn-CNP nano-fertilizer. Ferti-fortification using Zn-CNP nanocarrier certainly represents a new paradigm, viz. delivery of micronutrients in a ‘plant available’ form at the ‘right place’, ‘right time’ and ‘right dose’.

## Methods

### Field location and materials used

Field experiments were conducted at Agharkar Research Institute research farm, located at Hol, taluka Baramati, district Pune, Maharashtra (18° 31′ N, 73° 55′ E, average annual rainfall: 502 mm). The field trial was carried out during rabi season each year [winter, November to March 2011–14; average temperature 25.7 °C (see Supplementary Fig. S1); humid subtropical climate, soil type: clay loam]. Each of the trials was carried out on a different sub-plot which differed mainly in the soil zinc content. The ‘trial’ indicates the ‘study year’. The analysis of DTPA extractable (plant available) zinc from the soil collected from different sub-plots was carried out in Soil testing laboratory, Pune. Soil sampling was performed before the commencement of each trial. Each year, the soil collected was pooled, coning-quartering was performed, and representative samples were analyzed. Three samples per trial. The field trials #1 and #2 were carried out in plots with DTPA extractable zinc content 1.4–1.7 and 1.6–3.8 mg Kg^−1^ respectively. In trial #3 the soil zinc content was 1.2–1.4 mg Kg^−1^and in trial #4 it was only 0.3–0.4 mg Kg^−1^. Detailed soil analysis is providedin Supplementary Table S4.

For the study, two durum wheat (*Triticum durum* genotypes viz., MACS 3125 and UC 1114) were selected. MACS 3125 is a high yielding cultivar developed and released for cultivation in Maharashtra by Agharkar Research Institute, Pune; while UC 1114 is an exotic variety containing the GPC-B1 transcription factor^48^, which is responsible for high grain protein content.

Zn complexed chitosan nanoparticles (Zn-CNP) were synthesized as described in Deshpande *et al*.^31^. Briefly, chitosan (0.3 g) was dissolved in 1% v/v acetic acid and zinc sulfate (0.1 g%) was added. The solution was stirred continuously on a magnetic stirrer at ambient temperature (25 ± 3 °C) and 1 mL of sodium tripolyphosphate (1% TPP, v/v) was added dropwise to 25 mL chitosan solution to form Zn-CNP. For hardening, stirring was further continued for 20 min. The procedure for synthesis was scaled-up in batch mode to obtain 8 L particles in a single batch. Several batches were prepared to contain the zinc concentration (40 mgL^−1^, Zn-CNP1; and 4 mgL^−1^, Zn-CNP2).

### Treatments and experimental design

The seeding rate was 100 kg ha^−1^. The experimental plot was fertilized with N, P and K at the doses of 60, 40 and 20 kg ha^−1^ respectively before sowing. Further, after 21 days of sowing 60 kg ha^−1^ of nitrogen fertilizer was applied in the form of urea. The experiment was laid out in randomized complete block design in which treatments were main plots and cultivars were a sub-plot. Thus, each cultivar was sown in four replicate plots (each plot 1.5 m^2^, dimensions 1 m × 1.5 m). For each cultivar, there were 6 treatments and 4 replicates. Thus, during each season 48 plots (2 cultivars × 6 treatments × 4 replicates) were maintained. The foliar treatment groups were designated as:

Water (T0)

Urea (2% w/v) (T1),

Urea (2% w/v) + ZnSO_4_ 7 H_2_O (0.2%, ≡400 mg L^−1^ Zn) (T2),

Urea (2% w/v) + ZnSO_4_ 7 H_2_O (0.02%, ≡40 mg L^−1^ Zn (T3),

Urea (2% w/v) + Zn-CNP1, ≡40 mg L^−1^ Zn (T4), and

Urea (2% w/v) + Zn-CNP2, ≡4 mg L^−1^ Zn (T5).

In T2 the zinc applied in the form of foliar spray was 2000 g ha^−1^; whereas it was 200 g ha^−1^ in T3 and T4, and 20 g ha^−1^ in T5. The foliar application was initiated after anthesis, once-a-week, for up to five weeks (i.e. during the entire grain-development stage). The actual rate of application of foliar sprays was 200 mL/plot.

### Sample treatment and analyses

On maturity of the wheat plants, spike traits such as spikelet’s per spike, grains per spike and spike length were measured from five representative spikes from each replication. Thousand kernel weight (TKW) was obtained by measuring 250 grains from each plot harvest. Total grain weight obtained from each plot was converted to Kg per hectare and reported as grain yield. The protein content of the grain was determined by near infrared transmittance (NIT) on a Foss-Tecator 1241 (Foss, Hoganas, Sweden) instrument, which was calibrated using the Kjeldahl method (AACC approved method 46-12). One gram of wheat grains were weighed and transferred into glass tubes to analyze Zn and Fe content. Ten mL of concentrated three acid mixture (nitric acid, perchloric acid, sulfuric acid in 3:2:1 ratio) was added to each tube and left overnight at room temperature (28 ± 3 °C). The samples were then digested on a heater block at 125 °C for 2 h and 195 °C for 30 min. The digested samples were allowed to cool to ambient temperature (25 ± 3 °C) and diluted with 5% HNO_3_. After filtration through Whatman filter paper No. 1, Zn and Fe content in the acid-digested samples were determined using atomic absorption spectrophotometer (Analyst 800, Perkin Elmer, USA).

### Statistical analysis

The significance of the effects of treatments and their interactions on the reported traits and variety interaction was evaluated by analysis of variance (ANOVA) using CROPSTAT software while the Fisher’s least significant difference (LSD) test at 5% probability was used to compare treatment means.

### Data availability statement

All data generated or analyzed during this study are included in this published article (and its Supplementary Information files).

## Electronic supplementary material

Supplementary Information
