# Supplementary material for: Zinc use efficiency is enhanced in wheat through nanofertilization
Source: Sci Rep. 2018 May 1;8:6832. doi: 10.1038/s41598-018-25247-5 (PMC5931615; doi:10.1038/s41598-018-25247-5)
Supplement: Supplementary file 1 — Supplementary Information [file 41598_2018_25247_MOESM1_ESM.pdf]

Title: Zinc use efficiency is enhanced in wheat through nanofertilization

Authors names: Ashwin Dapkekar, Paresh Deshpande, Manoj D. Oak*, Kishore M. Paknikar* and Jyutika M. Rajwade*

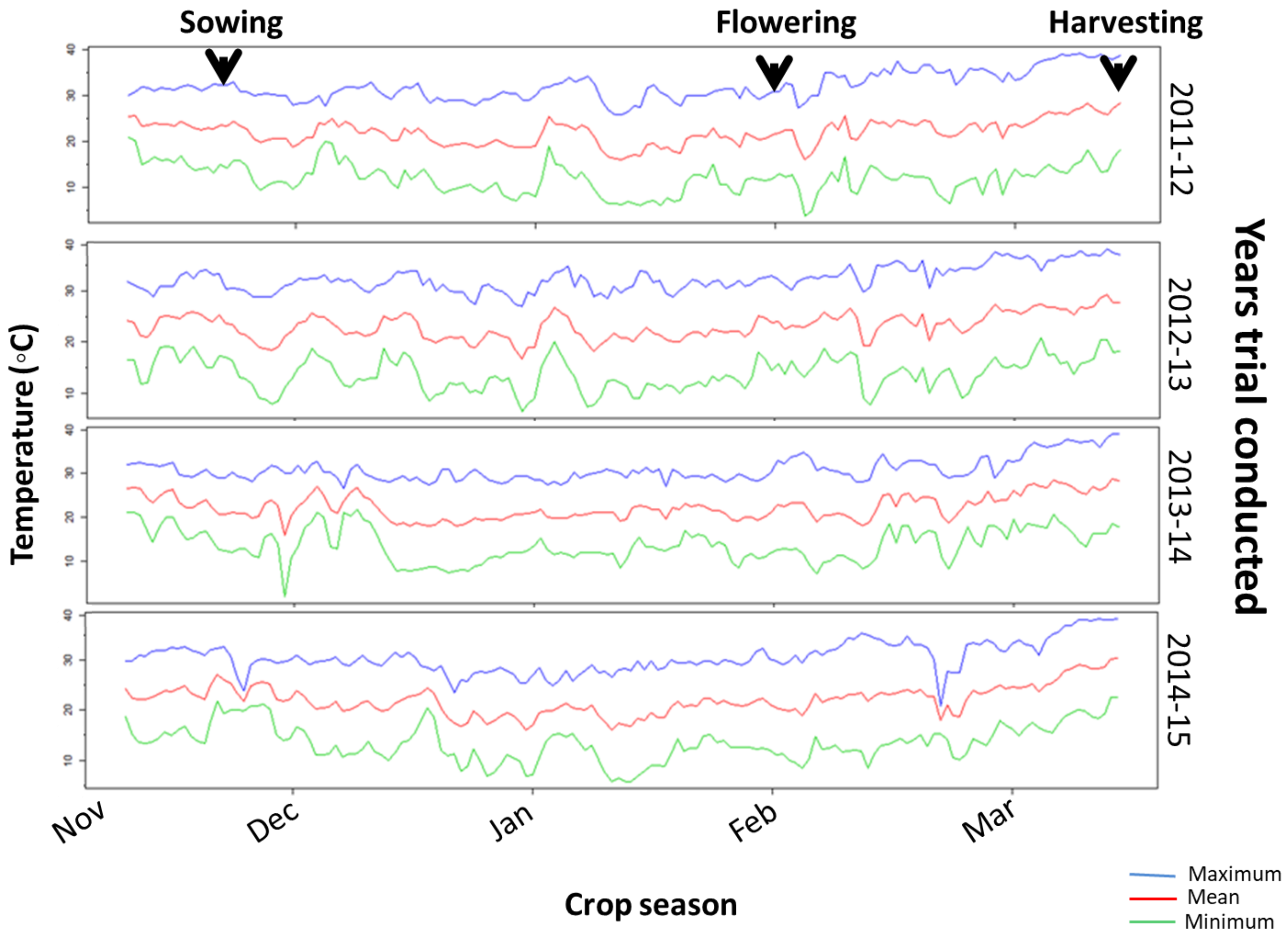

Figure S1. Variations in temperature during the cropping season over the four field trials

Trial	Cultivar	Treatment	Treatment Name	PC %	Zn content (µg/g)	Fe content (µg/g)
Trial 1 (2011-12)	MACS 3125	T0	Control	15.60A	42.18A	44.77A
		T1	Urea	17.25D*	52.59C*	54.71C*
		T2	U + $ZnSO_4$(400 mg L^{-1})	16.88C*	59.51D*	40.83B#
		T3	U + $ZnSO_4$(40 mg L^{-1})	16.93C*	51.66C*	42.80B#
		T4	U + Zn-CNP 1 (40 mg L^{-1})	16.45C*	52.97C*	43.60B#
		T5	U + Zn-CNP 2 (4 mg L^{-1})	17.00D*	50.19C*	42.97B#
	UC 1114	T0	Control	18.95a	37.86a	53.77a
		T1	Urea	20.03d*	43.97c*	49.65b#
		T2	U + $ZnSO_4$(400 mg L^{-1})	19.30c*	64.45e*	62.10d*
		T3	U + $ZnSO_4$(40 mg L^{-1})	19.33c*	56.57d*	59.35d*
		T4	U + Zn-CNP 1 (40 mg L^{-1})	19.98d*	55.92d*	68.95e*
		T5	U + Zn-CNP 2 (4 mg L^{-1})	20.10d*	46.19c*	53.15a
			$LSD_{0.05}$	0.6432	2.9199	3.7254
			$LSD_{0.01}$	0.8641	3.9227	5.0049
Trail 2 (2012-13)	MACS 3125	T0	Control	15.90A	53.46A	56.78A
		T1	Urea	16.18A	53.58A	57.52A
		T2	U + $ZnSO_4$(400 mg L^{-1})	17.80C*	68.95E*	62.74D*
		T3	U + $ZnSO_4$(40 mg L^{-1})	16.25A	55.01A	59.81C*
		T4	U + Zn-CNP 1 (40 mg L^{-1})	17.33C*	64.84D*	58.74C*

		T5	U + Zn-CNP 2 (4 mg L^{-1})	16.28A	55.87C*	54.19B#
	UC 1114	T0	Control	18.65a	56.39a	68.07a
		T1	Urea	18.63a	59.71c*	72.90d*
		T2	U + $ZnSO_4$(400 mg L^{-1})	19.20a	77.72e*	71.71c*
		T3	U + $ZnSO_4$(40 mg L^{-1})	17.47b#	62.12c*	70.19c*
		T4	U + Zn-CNP 1 (40 mg L^{-1})	20.25c*	70.97d*	72.57c*
		T5	U + Zn-CNP 2 (4 mg L^{-1})	18.43a	60.93c*	70.94c*
			$LSD_{0.05}$	0.8122	2.3603	1.8427
			$LSD_{0.01}$	1.0911	3.171	2.4756
Trial 3 (2013-14)	MACS 3125	T0	Control	15.40A	31.44A	40.88A
		T1	Urea	15.60A	32.79C*	43.82D*
		T2	U + $ZnSO_4$(400 mg L^{-1})	16.85E*	53.20F*	43.41D*
		T3	U + $ZnSO_4$(40 mg L^{-1})	16.15D*	38.43D*	42.38D*
		T4	U + Zn-CNP 1 (40 mg L^{-1})	16.83E*	45.38E*	44.42D*
		T5	U + Zn-CNP 2 (4 mg L^{-1})	15.85C*	31.05A	42.30C*
	UC 1114	T0	Control	18.28a	38.20a	62.47a
		T1	Urea	18.98c*	39.02a	66.48c*
		T2	U + $ZnSO_4$(400 mg L^{-1})	19.28d*	56.10e*	65.13c*
		T3	U + $ZnSO_4$(40 mg L^{-1})	19.13d*	41.32c*	64.87c*
		T4	U + Zn-CNP 1 (40 mg L^{-1})	19.28d*	48.17d*	65.60c*
		T5	U + Zn-CNP 2 (4 mg L^{-1})	18.75c*	37.79a	62.39a

			LSD $_{0.05}$	0.2783	1.0381	1.6911
			LSD $_{0.01}$	0.3739	1.3947	2.2719
Trial 4 (2014-15)	MACS 3125	T0	Control	14.00A	28.43A	39.83A
		T1	Urea	16.08D*	28.95A	41.22C*
		T2	U + $ZnSO_4$(400 mg L^{-1})	17.10E*	46.84F*	42.77C*
		T3	U + $ZnSO_4$(40 mg L^{-1})	14.98C*	34.24D*	40.04A
		T4	U + Zn-CNP 1 (40 mg L^{-1})	17.10E*	44.05E*	41.62C*
		T5	U + Zn-CNP 2 (4 mg L^{-1})	14.40C*	32.50C*	38.69A
	UC 1114	T0	Control	16.03a	31.09a	44.34a
		T1	Urea	17.45d*	32.30a	49.66d*
		T2	U + $ZnSO_4$(400 mg L^{-1})	18.20e*	48.45e*	46.11c*
		T3	U + $ZnSO_4$(40 mg L^{-1})	16.72c*	33.66c*	43.43a
		T4	U + Zn-CNP 1 (40 mg L^{-1})	18.00e*	45.95d*	47.10c*
		T5	U + Zn-CNP 2 (4 mg L^{-1})	16.28a	33.48c*	45.64c*
			LSD $_{0.05}$	0.3057	1.5683	1.7275
			LSD $_{0.01}$	0.4107	2.1069	2.3208

Table S1. The grain micronutrient (Zn and Fe) concentration and grain protein content of durum wheat as affected by application of Zn and urea in four trials. Values shown are means of 4 replicates. Means with same letter are similar at 5% significant level. * indicates significant increase, B# and b# indicates significant decrease. U denotes Urea, 2%.

**Trial**	**Cultivar**	**Treatment**	**Treatment Name**	**Yield (Kg ha^{-1})**	**TKW g**	**Spike length (cm)**	**Grains per spike**	**Spikelets per Spike**
Trial 1 (2011-12)	MACS 3125	T0	Control	5002A	43.00A	7.32A	40.4A	17.2A
		T1	Urea	4826A	41.20B#	7.1B#	39.2D#	16.8B#
		T2	U + $ZnSO_4$(400 mg L^{-1})	4944A	41.00B#	7.28A	36.8B#	16.5C#
		T3	U + $ZnSO_4$(40 mg L^{-1})	5122A	40.90B#	6.84B#	38.2C#	16.9B#
		T4	U + Zn-CNP 1 (40 mg L^{-1})	5347A	42.50A	7.45A	40.2A	17.2A
		T5	U + Zn-CNP 2 (4 mg L^{-1})	4744A	41.40B#	7.36A	40.8A	17.4A
	UC 1114	T0	Control	3389a	35.20a	7.10a	40.2a	17.4a
		T1	Urea	3199a	33.00b#	7.12a	38.2d#	16.8b#
		T2	U + $ZnSO_4$(400 mg L^{-1})	3135a	34.50a	7.23a	36.8c#	17.5a
		T3	U + $ZnSO_4$(40 mg L^{-1})	3647a	35.00a	7.15a	35.4b#	17.2a
		T4	U + Zn-CNP 1 (40 mg L^{-1})	3786a	35.60a	7.22a	38.4d#	17.4a
		T5	U + Zn-CNP 2(4 mg L^{-1})	2920a	34.90a	6.92b#	41.2*	17.2a
			LSD $_{0.05}$	585	1.1565	0.1353	0.8134	0.2707
			LSD $_{0.01}$	787	1.5537	0.1818	1.0928	0.3637

Trial 2 (2012-13)	MACS 3125	T0	Control	5287A	47.14A	7.65A	39.6A	17.3A
		T1	Urea	5693A	49.67C*	7.23B#	38.3D#	16.6C#
		T2	U + $ZnSO_4$(400 mg L^{-1})	4464B#	46.18A	7.03C#	34.4B#	16.0B#
		T3	U + $ZnSO_4$(40 mg L^{-1})	4464B#	49.50C*	7.25B#	35.75C#	16.8C#
		T4	U + Zn-CNP 1 (40 mg L^{-1})	5272A	47.66A	7.31B#	38.85D#	17.1A
		T5	U + Zn-CNP 2 (4 mg L^{-1})	6113C*	49.18C*	6.79C#	42.4*	17A
	UC 1114	T0	Control	3268a	34.51a	7.48a	44.45a	17.1a
		T1	Urea	2812b#	36.00c*	6.94b#	32.55b#	16.7b#
		T2	U + $ZnSO_4$(400 mg L^{-1})	2562b#	34.10a	7.27d#	37.3d#	17.7b*
		T3	U + $ZnSO_4$(40 mg L^{-1})	2394b#	36.78c*	7.31d#	33.2b#	17.1a
		T4	U + Zn-CNP 1 (40 mg L^{-1})	2817b#	33.70a	7.11c#	35.9c#	16.5c#
		T5	U + Zn-CNP 2 (4 mg L^{-1})	2945a	33.83a	6.88b#	41.4d#	16.5c#
			$LSD_{0.05}$	437	2.1437	0.1146	0.9607	0.485
			$LSD_{0.01}$	587	2.8799	0.1539	1.2906	0.6516
		T0	Control	4606A	52.80A	8.36A	40.8A	19.80A
		T1	Urea	4747A	53.50A	7.92B#	39.8B#	19.30A

Trial 3 (2013-14)	MACS 3125	T2	U + $ZnSO_4$(400 mg L^{-1})	4866A	53.30A	7.97B#	38.2C#	19.75A
		T3	U + $ZnSO_4$(40 mg L^{-1})	4908A	51.50A	8.04B#	38.6C#	19.70A
		T4	U + Zn-CNP 1 (40 mg L^{-1})	5195B*	54.00A	7.90B#	40.6A	19.63A
		T5	U + Zn-CNP 2 (4 mg L^{-1})	4990B*	51.50A	7.86B#	40.2A	18.75B#
	UC 1114	T0	Control	3244a	50.65a	7.54a	41.2a	20.50a
		T1	Urea	3381a	49.50a	7.02c#	40.2b#	19.30b#
		T2	U + $ZnSO_4$(400 mg L^{-1})	3249a	51.30a	7.67a	39.8c#	19.00c#
		T3	U + $ZnSO_4$(40 mg L^{-1})	3435a	50.50a	7.23b#	40b#	20.40a
		T4	U + Zn-CNP 1 (40 mg L^{-1})	3791b*	52.50b*	7.67a	41a	19.75b#
		T5	U + Zn-CNP 2 (4 mg L^{-1})	35.24a	50.75a	7.68a	41.5a	19.05c#
			$LSD_{0.05}$	339	1.4564	0.197	0.6371	0.4878
			$LSD_{0.01}$	455	1.9566	0.2647	0.8559	0.6553
	MACS 3125	T0	Control	5083A	40.87A	7.21A	41.4A	17.4A
		T1	Urea	5239C*	39.13C*	7.35B*	38.9B#	16.2B#
		T2	U + $ZnSO_4$(400 mg L^{-1})	4851B#	46.04C*	7.42B*	35.8D#	16.4B#
		T3	U + $ZnSO_4$(40 mg L^{-1})	4924B#	38.13B#	6.98B#	39.2B#	16.8B#

Trial 4 (2014-15)		T4	U + Zn-CNP 1 (40 mg L^{-1})	4898B#	41.18C*	7.1A	37.4C#	16.6B#
		T5	U + Zn-CNP 2 (4 mg L^{-1})	4808B#	42.92C*	7.05B#	40.4B	17.1A
	UC 1114	T0	Control	4650a	40.54a	7.3a	40.5a	16.9a
		T1	Urea	4458b#	36.48	7.10b#	41.2a	16.4b#
		T2	U + $ZnSO_4$(400 mg L^{-1})	4331c#	43.50	7.32a	38.8b#	16.4b#
		T3	U + $ZnSO_4$(40 mg L^{-1})	3993d#	43.50	6.98b#	39.6a	16.8a
		T4	U + Zn-CNP 1 (40 mg L^{-1})	4837e*	40.02	7.14b#	40.2a	16.7a
		T5	U + Zn-CNP 2 (4 mg L^{-1})	4864e*	37.68	7.02b#	38.8b#	17a
			LSD $_{0.05}$	103	0.3671	0.1156	0.8488	0.2655
			LSD $_{0.01}$	139	0.4932	0.1553	1.1404	0.3567

Table S2. Thousand kernel weight, grain yield and spike characteristics of durum wheat as affected by application of Zn and urea in four trials. Values shown are means of 4 replicates. Means with same letter are similar at 5% significant level. * indicates significant increase, # indicates significant decrease. PC % denotes grain protein content; grains per spike and spikelets per spike are actual numbers.

	**PC%**	**Zn**	**Fe**	**Yield**	**TKW**	**Spike length**	**Grains/spike**	**Spikelets/spike**
PC%	1							
Zn	0.4968	1						
Fe	0.7137	0.6413	1					
Yield	-0.7682	-0.3291	-0.7216	1				
TKW	-0.3540	-0.2646	-0.2061	0.4304	1			
Spike length	-0.2165	-0.2162	-0.2040	0.1544	0.6396	1		
Grains/spike	-0.1620	-0.4851	-0.2316	0.2157	0.2162	0.1865	1	
Spikelets/spike	0.0629	-0.2679	0.0666	-0.1294	0.6737	0.6848	0.3253	1

Table S3. Correlation coefficients for all the traits. PC denotes protein content, TKW denotes thousand kernel weight.

**Parameter**	**Trial # 1**	**Trial # 2**	**Trial # 3**	**Trial # 4**
Zn (mg kg^{-1})	1.45-1.70	1.66-3.83	0.34-0.40	1.22-1.45
P_2O_5 (kg ha^{-1})	67	67	20	54
Organic carbon %	0.68-1.21	1.12-1.18	0.59-0.73	0.72-1.01
pH	7.3-7.6	7.5-7.6	7.3-7.4	7.2-7.5
K_2O (kg ha^{-1})	306	622	277	331
Cu (mg kg^{-1})	2.18-2.89	1.86-2.26	2.27-2.65	2.20-2.42
Fe (mg kg^{-1})	2.92-4.89	2.27-3.98	11.74-14.31	3.20-4.62
Mn (mg kg^{-1})	13.01-26.58	10.26-11.14	51.61-63.13	14.22-20.2
Electrical conductivity (S cm^{-1})	0.27-0.50	0.36-0.51	0.26-0.33	0.28-0.52

Table S4. Soil characteristics. Soils were collected from the top 30 cm layer and analyzed according to the standard protocols described in Homer D. C. and Pratt P. F. (1961). Methods of Analysis for Soils, Plants and Waters. Davis: University of California, Davis.
